# Is Chytridiomycosis an Emerging Infectious Disease in Asia?

**DOI:** 10.1371/journal.pone.0023179

**Published:** 2011-08-16

**Authors:** Andrea Swei, Jodi J. L. Rowley, Dennis Rödder, Mae L. L. Diesmos, Arvin C. Diesmos, Cheryl J. Briggs, Rafe Brown, Trung Tien Cao, Tina L. Cheng, Rebecca A. Chong, Ben Han, Jean-Marc Hero, Huy Duc Hoang, Mirza D. Kusrini, Duong Thi Thuy Le, Jimmy A. McGuire, Madhava Meegaskumbura, Mi-Sook Min, Daniel G. Mulcahy, Thy Neang, Somphouthone Phimmachak, Ding-Qi Rao, Natalie M. Reeder, Sean D. Schoville, Niane Sivongxay, Narin Srei, Matthias Stöck, Bryan L. Stuart, Lilia S. Torres, Dao Thi Anh Tran, Tate S. Tunstall, David Vieites, Vance T. Vredenburg

**Affiliations:** 1 Cary Institute of Ecosystem Studies, Millbrook, New York, United States of America; 2 Department of Herpetology, Australian Museum, Sydney, New South Wales, Australia; 3 Zoologisches Forschungsmuseum Alexander Koenig, Bonn, North Rhine-Westphalia, Germany; 4 Department of Biological Sciences, University of Santo Tomas, Manila, Philippines; 5 National Museum of the Philippines, Manila, Philippines; 6 Department of Ecology, Evolution, and Marine Biology, University of California Santa Barbara, Santa Barbara, California, United States of America; 7 KU Biodiversity Institute, University of Kansas, Lawrence, Kansas, United States of America; 8 Biology Faculty, Vinh University, Vinh City, Nghe An, Vietnam; 9 Department of Biology, San Francisco State University, San Francisco, California, United States of America; 10 Department of Biology, Colorado State University, Fort Collins, Colorado, United States of America; 11 Faculty of Life Sciences, Southwest Forestry University, Kunming, Yunnan, China; 12 School of Environment, Griffith University Gold Coast Campus, Gold Coast, Queensland, Australia; 13 Faculty of Biology, University of Science-Ho Chi Minh City, Ho Chi Minh City, Vietnam; 14 Deptartment of Forest Resources Conservation and Ecotourism-Faculty of Forestry, Bogor Agricultural University Darmaga Campus, Bogor, West Java, Indonesia; 15 Museum of Vertebrate Zoology, University of California, Berkeley, California, United States of America; 16 Museum of Comparative Zoology, Harvard University, Cambridge, Massachusetts, United States of America; 17 Research Institute for Veterinary Science of Veterinary Medicine, Seoul National University, Seoul, Gyeonggi, South Korea; 18 Smithsonian Institution, Suitland, Maryland, United States of America; 19 Department of National Parks, Ministry of Environment, Phnom Penh, Cambodia; 20 Department of Biology, Faculty of Sciences, National University of Laos, Vientiane, Vientiane, Laos; 21 Kunming Institute of Zoology, Chinese Academy of Sciences, Kunming, Yunnan, China; 22 Department of Environmental Science, Policy, and Management, University of California, Berkeley, California, United States of America; 23 Department of Biology and Center for Biodiversity Conservation, Royal University of Phnom Penh, Phnom Penh, Cambodia; 24 Department of Ecology and Evolution, University of Lausanne, Lausanne, Vaud, Switzerland; 25 North Carolina Museum of Natural Sciences, Raleigh, North Carolina, United States of America; 26 Museo Nacional de Ciencias Naturales, Madrid, Spain; University of Bern, Switzerland

## Abstract

The disease chytridiomycosis, caused by the fungus *Batrachochytrium dendrobatidis* (Bd), has caused dramatic amphibian population declines and extinctions in Australia, Central and North America, and Europe. Bd is associated with >200 species extinctions of amphibians, but not all species that become infected are susceptible to the disease. Specifically, Bd has rapidly emerged in some areas of the world, such as in Australia, USA, and throughout Central and South America, causing population and species collapse. The mechanism behind the rapid global emergence of the disease is poorly understood, in part due to an incomplete picture of the global distribution of Bd. At present, there is a considerable amount of geographic bias in survey effort for Bd, with Asia being the most neglected continent. To date, Bd surveys have been published for few Asian countries, and infected amphibians have been reported only from Indonesia, South Korea, China and Japan. Thus far, there have been no substantiated reports of enigmatic or suspected disease-caused population declines of the kind that has been attributed to Bd in other areas. In order to gain a more detailed picture of the distribution of Bd in Asia, we undertook a widespread, opportunistic survey of over 3,000 amphibians for Bd throughout Asia and adjoining Papua New Guinea. Survey sites spanned 15 countries, approximately 36° latitude, 111° longitude, and over 2000 m in elevation. Bd prevalence was very low throughout our survey area (2.35% overall) and infected animals were not clumped as would be expected in epizootic events. This suggests that Bd is either newly emerging in Asia, endemic at low prevalence, or that some other ecological factor is preventing Bd from fully invading Asian amphibians. The current observed pattern in Asia differs from that in many other parts of the world.

## Introduction

Amphibian biodiversity is currently facing a severe crisis that has already caused declines in 42% of amphibian species and threatens as many as 32% with extinction [1], [2]. Remarkably, an emerging infectious disease, chytridiomycosis, is directly linked to the recent extinction or serious decline of hundreds of amphibian species, primarily anurans, and is increasingly proposed as a primary threat to amphibians [3], [4]. Chytridiomycosis, caused by the fungal pathogen *Batrachochytrium dendrobatidis* (Bd) [5], [6], [7], has been implicated as the causal agent in the greatest loss of vertebrate biodiversity due to disease in recorded history [3]. The severity of the current amphibian biodiversity crisis suggests that Bd is a fundamentally new challenge to amphibians compared to previous global and environmental changes that amphibians survived. The list of amphibian species driven to extinction by Bd is growing (>200 species) [3], [8] but there is still little known about the presence of this disease in Asia, an important area for amphibian biodiversity. This study is an attempt to evaluate the prevalence of Bd on a continental scale in Asia.

Bd proliferates in epidermal cells of post-metamorphic amphibians and in the mouthparts of tadpoles, and is capable of killing individuals rapidly via the disruption of skin function [9]. Like all chytridiomycota, Bd has an aquatic, flagellated zoospore stage. This zoospore infects the keratinized tissues of amphibians and then encysts, goes through asexual reproduction, and releases more propagules into the water [5], [10]. Because of this unique natural history, amphibian hosts with aquatic lifestyles are particularly susceptible to the pathogen [8]. Species with long aquatic larval stages or with mating or reproductive behaviors tied to lakes or streams tend to be more affected by Bd [11], [12], [13]. For example, one of the best-documented cases of Bd driving entire metapopulations of frog hosts to extinction is with the Sierra Nevada yellow-legged frog (*Rana sierrae*) and the southern mountain yellow-legged frog (*R. muscosa*) in western North America. In this system, Bd spreads into previously uninfected populations of frogs in a wave-like pattern driving populations to extinction only after average Bd infection intensity levels in a population reached very high pathogen density, or “load.” A pathogen load of 10^4^ zoospore equivalents per swab (Z_swab_), measured using a quantitative PCR assay specific for Bd [14], has been identified as a mortality threshold [12] and has also been called the “10,000 Zoospore Rule” [15]. Interestingly, in areas where Bd infection intensities remained low (below 10^3^ Z_swab_ per animal), populations were stable and did not crash [11]. It is still unknown why epizootic events occur in some areas and not in others. Many species are resilient to the pathogen and either do not become infected by it or do become infected and infection levels remain well below the *10,000 Zoospore Rule* minimum via mechanisms that are poorly understood (e.g. *Rana (Lithobates) catesbaeiana*, *Xenopus leavis* [8], *Litoria wilcoxi* [16]). Species that are non-susceptible to Bd are presumably protected by one or more of a number of factors: poor abiotic growing conditions for Bd [17], immunological response [18], low virulence of Bd strain, or bacterial epibiotic symbionts [19].

Nearly 12 years after Bd was first described [10] there is still very little known about the mechanisms of large scale movement of Bd. This is, in part, due to the incomplete understanding of the global distribution of Bd. On a continental scale, several non-susceptible frog species have been proposed as vectors of transmission. They include the American bullfrog (*Rana catesbeiana*) [20], [21], [22], the African clawed frog (*Xenopus laevis*) [22], and several species of frogs in the genus *Telmatobius* [23]. These species do not succumb to the disease, and the American bullfrog and African clawed frog are highly invasive, traded (alive) in enormous quantities throughout the world [20], and have in some cases been linked to the distribution of the pathogen in other species [20]. However, of the two best-documented examples of Bd behaving like an epidemic wave (i.e. Central America and California) no animal or environmental reservoir was found to be responsible for the rapid regional movement of the pathogen [7], [12]. In both cases, the disease spread rapidly and travelled in a linear, wave-like manner, suggesting that the pathogen is emerging and not just becoming more apparent due to some other environmental change [7], [12].

Research on Bd in Asia as a whole has been largely neglected relative to other continents such as North America, Central and South America, Europe, and Australia. Asia is a region of particular interest for Bd because there is high amphibian diversity, and thus great potential for species losses. The only reports of amphibians with Bd come from in Japan [24], [25], South Korea [26], China [27], and Indonesia [28]. Other studies have looked for Bd in Asia but have failed to find it [29], [30]. Thus far, there have been no reports of massive die-off from a Bd epidemic. The most extensive study, which took place in Japan, found Bd at low prevalence in field samples from native frogs and salamanders, but higher prevalence among non-native species of amphibians (e.g. *Rana catesbeiana*) [25]. On the mainland of Asia there have been reports of Bd from South Korea (three species infected including two native frogs and non-native, *R. catesbeiana*) [26], and in Yunnan Province, China (four species infected including three native frogs and non-native *R catesbeiana*) [27]. However, despite these reports, there are little or no data throughout most of Asia, and where there are data the prevalence is low compared to the pattern of disease emergence seen in other parts of the world. Infection intensity (Z_swab_) on individual hosts has been proposed to be a key component in predicting whether Bd presence causes chytridiomycosis and death [11], [12], yet only one study provides these data from Asia [27] and none of the infection levels reported reached the mortality threshold of the *10,000 Zoospore Rule*.

**Figure 1:**
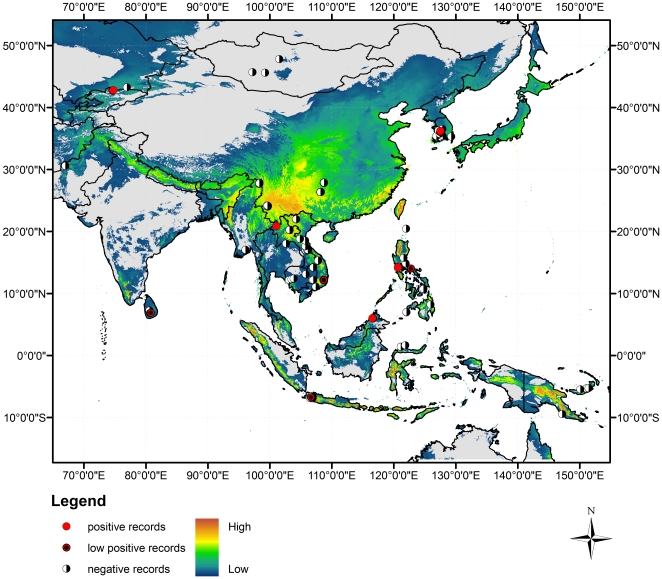
Map of predicted and observed *Batrachochytrium dendrobatidis* distribution in Asia. Map of Asia and Papua New Guinea showing Maxent predicted probability of *Batrachochytrium dendrobatidis* (Bd) from low to high environmental suitability [28]. Sample localities from field surveys are shown as black and white, red with black dot, and red circles indicating the highest level of Bd infection found. A high-resolution image is available in Supporting Information.

Recently a global species distribution model (SDM) using a presence-only method was developed for Bd [31] supplementing regional predictions [32], [33], [34]. This SDM model was developed in Maxent based on 365 worldwide Bd presence localities compiled in the disease records database (www.bd-maps.net) and bioclimatic variables matching the physiological limits of Bd in both laboratory [17] and field studies [35], [36]. Rödder et al. [31] predicted that the bioclimatic conditions in Asia permits considerable potential for Bd to emerge. Particularly, the SDM highlights potentially suitable areas in southern and southwestern China, the northern Philippines, western Myanmar, northern and central Vietnam, central Indonesia, and Papua New Guinea (Figure 1). The areas highlighted by the SDM (shown as highly suitable for Bd) provide suitable climatic conditions for the establishment of the pathogen given the presence of sufficient host species and dispersal capacities. However, this SDM did not include any locality data in Asia as no records from this area were available when it was created.

We propose that the lack of enigmatic mass die-off in Asia signals three possible scenarios: 1) Asia is a region of emerging Bd and the next few decades could present similar epidemic decline and extinction events as has been documented in Australia, California, the Caribbean, and Central and South America, or 2) Bd is endemic to Asia and therefore amphibians and Bd share an evolutionary history such that large-scale epidemics and extinctions are unlikely, or 3) there is a fundamental difference in epidemiology of disease dynamics on amphibian hosts in Asia where infected animals never surpass the load set by the *10,000 Zoospore Rule* and thus do not succumb to chytridiomycosis. In this study, we assess the evidence for these scenarios. Finding a spatial distribution of Bd in Asia that is consistent with other sites of disease epidemics would support to scenario one. Scenario two, proposed recently [25], is based on the unpublished record of an apparently Bd-infected Japanese giant salamander (*Andrias japonicus*) from a museum specimen collected in 1902 and preliminary molecular data that identified a unique, basal haplotype infecting *A. japonicus* [25]. To definitely validate this scenario would require further genetic and/or museum specimen record examination and follow up field studies to study the dynamics of Bd in wild populations. Confirmation of scenario three will also require further detailed epidemiological, physiological, and genetic analyses.

The general lack of understanding of Bd in Asia and the predicted high climatic suitability for spread motivated the desire to gather data on Bd in Asia. This study seeks to determine the potential for Bd to spread through Asian amphibian populations and to identify particular regions where Bd may be emerging. This study also aims to assess the degree to which current species distribution models are able to predict the current distribution of Bd.

## Methods

### Ethics Statement

This research was conducted under IACUC number A8-006 issued by San Francisco State University to VTV. Local sampling permits were obtained from each of the countries surveyed in this study. No animals were harmed by research while collecting swab samples.

### Field surveys

Our field surveys for Bd in Asia and adjoining Papua New Guinea were widespread and opportunistic. Most samples came from the wild except 20 samples of *Hoplobatrachus rugulosus* were collected from two frog farms in Vientiane, Laos (a commercial farm called Aquaculture Breeding Training Center and an unnamed private farm). A total of 3363 samples were collected for use in this study, which were spread among 298 sampling localities in 15 countries (Table 1). Samples were collected over a period of 9 years between 2001 and 2009.

**Table 1:** Infection status with *Batrachochytrium dendrobatidis* by country.

Country	Samples		Bd Status		Bd Status including low infection positives
		High	infection positive	Low	infection positives
	No.	No.	% High positive(95% Credible Interval)	No.	% Low positive(95% Credible Interval)
Cambodia	384	0	0 (0–0.95)	0	* no change
China	256	0	0 (0–1.43)	0	* no change
Indonesia	797	3	.25 (0.14–1.09)	8	1.38 (0.72–2.36)
Kazakhstan	4	0	0 (0–45.07)	0	* no change
Kyrgyzstan	9	9	100 (69.15–99.75)	0	* no change
Laos	551	4	0.73 (0.29–1.84)	0	* no change
Malaysia	111	1	0.90 (0.21–4.87)	1	1.80 (0.56–6.30)
Mongolia	23	0	0 (0.11–14.25)	0	* no change
Myanmar	62	0	0 (0.04–5.69)	0	* no change
Pakistan	5	0	0 (0.42–45.93)	0	* no change
Papua New Guinea	73	0	0 (0.03–4.86)	0	* no change
Philippines	412	33	8.01 (5.77–11.04)	1	8.25 (5.96–11.28)
South Korea	29	1	3.45 (0.82–17.22)	1	6.9 (2.11–22.07)
Sri Lanka	117	0	0 (0.02–3.10)	10	8.55 (4.38–14.51)
Vietnam	530	0	0 (0–0.69)	7	1.32 (0.65–2.70)
**Total**	**3363**	**51**	**1.52 (1.15–1.98)**	**28**	**2.35 (1.89–2.92)**

All countries sampled in this study and status of amphibian infection with *Batrachochytrium dendrobatidis*. Positive samples were divided into “low infecion” and “high infection” positives with low infection defined as samples with corrected genomic equivalent (Z_swab_) values <1 but >0. Percent positives are given in the columns along with the 95% Bayesian credible intervals.

### Lab analysis

Infection status of samples was determined by either histology or quantitative real time quantitative PCR (qPCR) of skin swabs [14], [37]. Histology was conducted on 826 samples from Cambodia and Indonesia at James Cook University or Sihanouk Hospital and the Royal University of Phnom Penh. Samples were fixed in 10% formalin, stored in 70% ethanol, and examined by histology within a year of collection. Histology examined 3 mm^2^ sections of amphibian skin that were removed from the animal's pelvic patch [38]. Skin samples were embedded into paraffin and cut into 5 µm strips, then stained with Periodic acid-Schiff (PAS). Six skin ribbons per individual were examined for signs of infection by Bd, with known positive slides as a comparison.

The remaining samples were tested by PCR (n = 844), or qPCR (n = 1693) [14], [37] in three laboratories (Supporting Information Table S1). Skin swab samples were swabbed in the field using a standardized protocol [37]. A synthetic swab was used to sample each specimen by swabbing each individual with 30 strokes: each hindfoot was swabbed five times on the toe webbing, each thigh was swabbed five times, and each side of the ventral abdomen was also sampled 5 times [12]. Swabs were stored dried in 1.5 ml tubes until processed in the lab. For samples ran using regular PCR (Pisces Molecular; Boulder, Colorado), samples were pooled into groups of 8 samples prior to PCR analysis and run in triplicate with positive and negative controls. For qPCR, swabs were extracted using PrepMan ultra sample preparation reagent (Applied Biosystems, Foster City, CA) and run in duplicate [37]. Negative controls were included on each test plate. Quantitative estimates of Bd zoospore loads (Z_swab_) were generated using standard samples of known Bd quantity on each plate. All standards were obtained from the Australian Animal Health Laboratory (courtesy of A. Hyatt). To estimate the total number of zoospores on each swab, we calculated a corrected GE score by multiplying raw GE scores by 80 to correct for dilution from extraction and PCR, this value is referred to as a Z_swab_ score [12]. Samples were defined as “positive” if the Z_swab_ was ≥0 however those samples with a Z_swab_<1 but >0 are categorized as “low infection” positives whereas Z_swab_≥1 are “high infection” positives to distinguish between these two types of positives. Histological samples were also classified as “low infection positives” if zoospore structures were faded or non-traceable.

### Data analysis

Bd infection prevalence was calculated for each country and a 95% Bayesian credible interval for infection prevalence was calculated for each country. Because of minimal knowledge of Bd prevalence in Asia we assumed a uninformative priors using a uniform beta distribution for infection prevalence [39], [40]. Credible intervals were calculated using the inverse of the cumulative distribution function of the beta distribution in R (i.e. qbeta) [41] where the shape 1 parameter was specified as μ+1 and. the shape 2 parameter was specified as N-μ+1, where μ is the number of positive samples and N was the total sample size. All analyses were executed in R.

### Species distribution model comparison

The species distribution model for Bd was previously generated by Rödder et al. [31] using Maxent [42] with presence-only localities of Bd from Europe, Australia, and the Americas [31]. Here we provide a more detailed map, focusing on Asia, showing the predicted probability of environmental suitability for Bd from the worldwide logistic output of Rödder et al. All but 4 of the 298 Asian localities sampled in this study were included in this comparison. The probability of environmental suitability derived from the Maxent model can be interpreted as the likelihood that Bd may establish at a specific site given the environmental conditions, that there is a sufficient number of host species, and that the site is accessible for the pathogen and hence represents its potential distribution [43]. To test its predictive performance, Maxent allows the computation of a modified version of the area under the receiver (AUC) operating characteristic curve [44] scores derived from presence records and random background data accounting also for prevalence of the area predicted as suitable [42]. These AUC scores summarise the trade-off between false-positives and false-negatives across all possible thresholds throughout the probability function.

We chose AUC as validation method since it is easily interpretable, non-parametric, and threshold-independent. Furthermore, in this specific case this method is superior to other methods e.g. logistic regressions or site occupancy models [45] because these other methods require a much higher number of positive observations to compensate potential problem caused by zero-inflation. Furthermore, logistic regression analyses and site occupancy models focus on a species' realized distribution, whereas the focus of the present work is Bd's potential distribution, which can be better captured using presence-only or presence/pseudo-absence approaches [46] AUCs were computed using (1) all positive records from this study (AUC_relaxed_) and (2) only high infection positives (AUC_strict_). As background area, we used a minimum rectangular polygon enclosing all tested sites and 10,000 random records as pseudo-absences. All computations were conducted internally in Maxent using the command line functions. An AUC validation method was selected here over Kappa statistics and True Skills Statistics (TSS) because these other methods require true absence records. In Asia, true absences for Bd are difficult to compile since to date, it is not clear whether Bd is still emerging and just not present at suitable sites due to limited accessibility or absent due to unsuitable environmental conditions. Only the latter case may allow a reliable assessment of the predictive performance of SDMs.

In addition to calculating AUC, we regressed the Maxent predicted probability of Bd presence against our qPCR Bd load results to see if the model predicted disease intensity. Our dataset included qPCR data from 9 of the 15 positive localities. These load data were averaged by locality. Linear regressions were performed across all localities and on positive localities only.

## Results

Between 2001 and 2009, we tested 3363 amphibians from Asia and Papua New Guinea for infection with Bd. Our samples spanned 15 countries, approximately 36° latitude, 111° longitude, and over 2000 m in elevation. Overall, our survey found that infection prevalence was very low at the 298 localities (Table S1). Specimens positive for Bd did not follow a clear geographic pattern and are scattered widely across Asia (Fig. 1). Of the 15 countries sampled, the Philippines, Kyrgyzstan, Laos, Indonesia, Malaysia, and South Korea were the only countries with high infection positives. Of the 3363 samples tested, 51 were positive with high infection for Bd (1.52%; 1.14–1.97, 95% credible interval) and an additional 28 were low infection positives (Table 1). The highest prevalence was found in Kyrgyzstan with 100% (n = 9) infection prevalence but prevalence in other countries were all below 10%. Overall, 2.35% of our samples were positive for Bd infection.

Two amphibian orders (Anura and Caudata) were sampled in this study although most samples came from anura (n = 3315 vs n = 48; Table S2). No infected salamanders were found in this study, but sampling of salamanders was limited. Among the frogs, infection with Bd was taxonomically variable with infected samples from 6 of 13 families (Table 2; 8 of 13 when we include low infection positives) and from 8 of 59 genera (15 of 59 when we include low infection positives; Table 3). We discovered Bd infection in 14 of 242 species (31 of 242 when we include low infection positives), but the number of infected species is likely to be higher because several positive samples came from animals of currently unresolved taxonomic status (e.g. *Philautus* sp. and *Limnonectes* sp., Table 2). Of the resolved species, the most commonly infected species was the endemic Philippine ranid, *Hylarana similis* (27/30 Bd infected, Table 3). Low infection positive infections were generally found in countries that also contained high infection positives but Sri Lanka and Vietnam contained isolated low infection positive samples but no high infection positives (Table 2). The conservation status Bd positive species tended to be of low concern (LC) or no threat (NT) (Table 1), but among the low infection positive samples, several are listed as endangered (EN) or vulnerable (VU) by the IUCN including *Huia masonii* and the rhacophorids *Pseudophilautus alto*, *P. silus*, and *Polypedates eques* (Table 3).

**Table 2:** Location of species infected with *Batrachochytrium dendrobatidis*.

Country	Location, elevation	Date	Family	Genus	Species	IUCNstatus	Pos/Tot
Kyrgyzstan	42.728°N, 74.648°E	May-05	Bufonidae	*Pseudepidalea*	*pewzowi×turanensis*		1/1
	42.681°N, 74.657°E	to			*pewzowi*		6/6
	42.728°N, 74.648°E; 42.794°N, 74.76°E	Aug-05			*turanensis*	LC	2/2
Luang Namtha, Laos	20.868°N, 101.055°E, 1000 m	Mar-07	Megophryidae	*Leptolalax*	sp.		2/22
			Ranidae	*Odorrana*	*chloronota*	NT	1/15
			Rhacophoridae	*Philautus*	sp. (Laos)		1/5
Mt Kinabalu, Malaysia	6.009°N, 116.543°E, 1563 m	Jan-06	Rhacophoridae	*Philautus*	sp. (Malaysia)		1/5
Mt. Palay Palay-Mataas na Gulod NP, Philippines	14.232°N, 120.658°E, 330 m	2006–2007	Dicroglossisae	*Limnonectes*	*woodworthi*	LC	4/9
				*Occidozyga*	*laevis*	LC	2/2
			Ranidae	*Hylarana*	*similis*	NT	27/30
Sinpyeong, South Korea	36.227°N, 127.505°E	Jun-06	Hylidae	*Hyla*	*japonica*	LC	1/1
Desa Kabiraan, Indonesia	†, 1949 m	Jan-05	Ranidae	*Hylarana*	*chalconota*	LC	1/1
Loka, Indonesia	5.44611°S, 199.92278°E†, 897 m	Jan-05	Dicroglossidae	*Limnonectes*	sp.		1/8
Mamasa, Indonesia	2.94145°S, 199.27788°E†, 897–1102 m	Jan-05	Ranidae	*Hylarana*	*chalconota*	LC	1/2

*Batrachochytrium dendrobatidis* positive samples by locality and species including the number of infected samples and sample size. Conservation status is provided as International Union for the Conservation of Nature (IUCN) status except for species with unresolved taxonomic status.

† Exact locality unknown.

**Table 3:** Samples with “low” infections of *Batrachochytrium dendrobatidis*.

Country	Location, elevation	Date	Family	Genus	Species	IUCN status	Low+/Total
Indonesia	NA, 1173 m	5-Oct	Bufonidae	*Ingerophrynus*	*celebensis*	LC	1/14
	−6.74126111°N 106.649628°E, 1000 m	6-Jul	Ranidae	*Huia*	*masonii*	VU	2/58
	NA, 1949 m	5-Nov		*Hylarana*	*chalconota*	LC	1/32
	NA, 897	5-Nov			*macrops*	NT	1/4
	6.74126111°N 106.649628°E, 1000 m	6-Jul		*Odorrana*	*hosii*	LC	4/54
Malaysia	6.00861111°N, 116.542769°E, 1563 m	10-Jan	Dicroglossidae	*Limnonectes*	*kuhlii*	LC	1/11
Philippines	14.03936667°N, 122.78655°E	2006–2007	Ranidae	*Hylarana*	*luzonensis*	NT	1/28
South Korea	36.05151667°N, 127.477117°E	6-Jun	Bombinidae	*Bombina*	*orientalis*	LC	1/8
Sri Lanka	6.84333333°N, 80.6777778°E, 1638 m		Dicroglossidae	*Fejervarya*	*limnocharis*	LC	3/7
	6.884558333°N, 80.8013611°E, 1878 m		Ranidae	*Hylarana*	*temporalis*	NT	1/18
	6.84333333°N, 80.6777778°E, 1638 m		Rhacophoridae	*Pseudophilautus*	*alto*	EN	1/26
	6.84333333°N, 80.6777778°E, 1638 m				*silus*	EN	1/12
	6.84333333°N, 80.6777778°E, 1638 m				*sordidus*	NT	1/11
	6.84333333°N, 80.6777778°E, 1638 m			*Polypedates*	*eques*	EN	1/10
	6.84333333°N, 80.6777778°E, 1638 m		Nyctibatra-chidae	*Lankanectes*	*corrugatus*	LC	2/7
Vietnam	12.18644444°N, 108.714861°E, 1878 m and 12.19313889°N, 108.7112694°E, 1864 m	8-May	Megophryidae	*Ophryophryne*	sp.		2/35
	12.18644444°N, 108.714861°E, 1627 m and 12.19261111°N, 108.711583°E, 1886 m	8-May	Rhacophoridae	*Philautus*	*gryllus*	DD	5/39

Samples designated as “low infection” positives for *Batrachochytrium dendrobatidis* by site and species including number of low infected positive samples and total sample size. Conservation status is provided as International Union for the Conservation of Nature (IUCN) status.

Individual host Bd infection intensity (Z_swab_) results from the qPCR assay were low (Table 4). All of the Bd positive countries had average Z_swab_ scores below the epidemic threshold, called the *10,000 Zoospore Rule* [15], determined in other species (e.g. 10^4^ Z_swab_) [12] (Table 4). The Philippines had the highest total number of positively infected animals and the highest mean Z_swab_ scores (mean = 325.814±107.07 (SE)) while Indonesia had the lowest Z_swab_ scores (Table 4).

**Table 4:** Quantitative pathogen loads on positive samples.

Country	High infection positive (N)	Low infection positive (N)
Indonesia	1.607±0.337 (3)	0.355+0.126 (2)
Laos	65.63±49.768 (4)	NA
Malaysia	3.019±NA (1)	7.5e-05±NA (1)
Philippines	325.814±107.065 (33)	NA
Vietnam	NA	0.751±0.248 (7)
Mean	268.84±87.97 (41)	0.59±0.19 (10)

Mean corrected genomic equivalents (Z_swab_) scores and standard errors for countries with samples that tested positive for *Batrachochytrium dendrobatidis* by qPCR. Low infection positives were defined as samples with corrected genomic equivalents less than 1 or by ambiguous histology results. Sample size of positives from each country are given in parentheses.

### Model comparison

Our field sampling efforts found that only 5.03% (15 of 298) of our localities had one or more samples that tested positive for Bd. When low infection positives are excluded, only 2.34% (7 of 298) of our sampling localities had samples with high infection positives. We analytically compared the SDM predicted environmental suitability with observed field pathogen presence at 294 localities in Asia and Papua New Guinea. We excluded 4 localities due to inexact coordinates; all four sites were Bd negative. All but three localities had Maxent suitability scores above the minimum training presence threshold (98.98%) and 203 scores above the 10 percentile training omission threshold (68.81%) as reported by Rödder et al. [31] suggesting that the environmental conditions at the study sites are generally favourable for Bd. AUC scores internally computed in Maxent revealed a good discrimination ability of the model (AUC_strict_ = 0.803; AUC_relaxed_ = 0.881) meaning that the 15 localities with Bd positive samples were found in environmental conditions consistent with SDM predictions. However, despite environmental suitability throughout much of the sampled areas of Asia, our surveys did not find Bd in most sites predicted to be suitable (Fig. 1).

Our analyses of Maxent predicted probabilities and Bd infection load found no correlation either with the full dataset (many negative localities, r^2^<0.001, *P* = 0.36) or with only positive localities (r^2^ = 0.014, *P* = 0.32).

## Discussion

Amphibian diversity in Asia encompasses some of the most diverse amphibian assemblages across all three orders (Anura, Caudata, Gymnophiona) and extant species include members representing both trunk and crown diversity for Class Amphibia. Worldwide amphibians are rapidly disappearing with more than a third of the species threatened with extinction. Causes are many but the chytridiomycosis pandemic has been implicated in several severe cases of declines in protected, undisturbed areas [5], [7], [12], [23]. The extreme virulence of this pathogen and its ability to drive multiple host species (>200 estimated) to extinction is unmatched in its threat to vertebrates [3]. Bd has been linked with population declines and species extinctions on every continent that harbours amphibians except Asia [8]. We undertook the most extensive survey of Bd in Asia to date to determine if Bd is a threat to amphibians in Asia.

Despite extensive amphibian sampling (n = 3363) throughout Asia and Papua New Guinea, our opportunistic study found very low Bd-infection prevalence (2.2% positives) in frogs and salamanders (caecilians were not encountered). All 74 of the Bd-positive samples were collected from frogs, but sampling of salamanders was very limited and warrants expanded sampling in future studies. With the exception of a few countries, our 95% credible Bayesian intervals indicate that prevalence is indeed low in most Asian countries (Table 1). Our results are consistent with previous studies in Asia that have reported low prevalence of Bd (S. Korea [26], Indonesia [28] and China [27] and Japan [25]) or that did not detect Bd (Hong Kong [29], Thailand).

Analysis of samples by qPCR allowed us to assess the relative pathogen load on each sample to determine if Bd infection intensities were nearing epidemic levels as has been documented in areas where Bd is an emerging disease causing host population collapse [12]. The Z_swab_ scores that we report here were all well below the mortality threshold previously reported in other anuran species [12] and there was not correlation between pathogen load and Maxent predicted probability. We did however discover one site in the Philippines where we found relatively high mean Z_swab_ scores, 300+ (Table 4). However, surveys at each locality were conducted once so we cannot determine if prevalence or Z_swab_ scores are increasing or decreasing.

The SDM by Rödder et al. [31] suggested that the environmental conditions at all sampled sites are suitable for Bd (Fig. 1). However, when comparing our field Bd infection prevalence with the model predictions, of 294 sampling localities, only 15 (5.03%) proved to be positive for Bd. These results suggest at the time of our surveys that the distribution of Bd was much less widespread than may be expected based on environmental suitability alone. As stated above, it is necessary to distinguish between the absence of Bd due to unsuitable environmental conditions and its absence due to limited dispersal or establishment abilities which is beyond the limits of SDM. Furthermore, our results suggest that although Bd may be present in one population it may be absent in other populations in close proximity. Similar patterns were also reported from Switzerland [47] suggesting that small scale variations may occur, which cannot be explained by differences in climatic conditions but are probably due to dispersal or persistence capacities of Bd. The new Bd records presented in this paper suggest that positive Bd records occur in areas that are environmentally consistent with Bd-localities found elsewhere in the world. However, most areas of Asia predicted by SDM to be suitable for Bd were not found to have positively infected samples in this study.

We propose three hypotheses that could explain why Bd is not currently responsible for amphibian declines in Asia: *1) Bd has not yet emerged, 2) Bd is endemic and shares an evolutionary history with native host species, 3) Bd emergence is inhibited due to abiotic or biotic reasons.* It is possible that despite suitable hosts and environments, Bd has not yet emerged in Asia. The SDM clearly predicts that suitable conditions are widespread throughout Asia [31], yet although we sampled in many of those areas, we found very few infected amphibians. Thus the low prevalence that we and others report in Asia could be the leading edge of multiple introductions. However, the geographic distribution of Bd-positive samples in our study was widespread but did not indicate a clear geographic pattern or directionality. An emerging disease should exhibit some of the same geographic patterns documented in other pandemic areas and follow a linear, wave-like pattern of distribution [12], [48], although multiple, simultaneous introductions are possible. Sites positive for Bd spanned from Indonesia to South Korea and from Kyrgyzstan to the Philippines (Fig. 1). Kyrgyzstan had the highest infection prevalence but few samples (100%, n = 9). The highest number of infected amphibians was in the Philippines (8.01%, n = 33). Thus, the spatial distribution of Bd in Asia on a continental scale does not reflect a wave-like geographic spread that would indicate an invading pathogen as was reported in Central America [48] and California [12]. These other studies showed that Bd invaded into naïve amphibian host populations where Bd prevalence went from zero to 100% and was followed by massive population and species collapse. High prevalence alone did not lead to collapse [11], but only occurred after individual Bd infection levels reached a mortality threshold, surpassing the *10,000 Zoospore Rule* (Z_swab_>10^4^) [12], [15]. Thus the current spatial distribution of Bd does not indicate an actively spreading disease epidemic that should exhibit a wave-like pattern.

Our second hypothesis that Bd is endemic to Japan or Asia would imply that endemic species that share an evolutionary history with Bd are resistant. A museum record of a potentially Bd-infected salamander in Japan as early as 1902 [25] suggests that Bd may not be new to Asia and that the pattern of disease emergence is different in Asia compared to Central America and California. If this were the case, we would expect to see higher prevalence of Bd. The low prevalence values that we report for Asia could not be sustained without continued reintroduction of the pathogen from a reservoir and would indicate that the disease is not self-sustaining [49]. A more thorough historical analysis of museum records through space and time is needed to determine the historical presence of Bd throughout the region but our preliminary study indicates that if Bd is endemic to Asia, epidemiologically it does not explain the lack of declines there. It will also be important to compare the genetic strains of Bd found in Asia with those found at sites associated with mass die-offs to determine if strain difference in the pathogen can explain the difference in disease prevalence in Asia [50], [51].

Our last hypothesis encompasses the many potential factors that may explain possible fundamental differences in Asia but are difficult to detect on a continental scale. One factor that could be important in Asia is the presence of symbiotic skin microbes that in other Bd-infected amphibian hosts have been important in reducing the severity of the chytridiomycosis [19]. There are no reports currently about the epibiotic microbiota of Asian amphibians and whether they may protect hosts from Bd. We propose that this is an area that deserves attention given the strikingly different pattern of Bd prevalence in Asia compared to other areas.

Many more data are needed from Asia to fully assess the threat of Bd to that regions' amphibian species. Although Bd appears widely distributed throughout Asia, there is limited evidence that Bd is a novel pathogen presenting an immediate threat to amphibian populations in Asia. Thus far, no epidemics have been reported, but that may change as the disease emerges or as conditions change (e.g. climate change, general biodiversity loss). We recommend long-term amphibian population monitoring throughout Asia in order to detect any amphibian population declines, particularly at sites where Bd has already been detected. We propose that the most likely region from our study where an epidemic may emerge is in the Philippines. At one site we found the highest number of infected samples than anywhere else in Asia and the Z_swab_ values were higher than in any other area we sampled in Asia. Sri Lanka was also notable partly because of the higher Bd prevalence documented but also because the high levels of biodiversity and endemicity of amphibian species found there make conservation a very high priority. We document high infection prevalence in Kyrgyzstan as well but samples were extremely limited in number therefore it is difficult to determine the significance of our findings there at this time. We suggest that an assessment of the susceptibility of Asian amphibians, especially montane species, to Bd infection and disease development should be carried out via laboratory Bd infection trials of wild-caught Asian species as well as through repeated field surveys. It will be important to monitor the status of areas where positives are detected to see if prevalence and infection intensity rise. If the *10,000 Zoospore Rule* [15] does apply broadly to amphibians infected with Bd, then quantitative PCR should help identify sites where mortality is imminent and thus those sites should be carefully studied. Our study revealed only one site, located in the Philippines, where infection intensities were much higher than anywhere else, yet they were still well below the threshold (∼300 Z_swab_ vs 10,000 Z_swab_; no dead or dying animals were found during the surveys). Because the epidemiology of Bd appears to be different in Asia compared to other regions of Bd emergence, insights gained in the region may contribute significantly towards resolving the origin and mechanism of Bd emergence globally.

The low prevalence of Bd we document in Asia and a comparison of our field results with SDM predictions supports either our hypothesis 1, i.e. that Bd has not emerged yet in Asia and is following an unusual spatial distribution, or, hypothesis 3, that the divergent field and model prediction data that we present here may indicate that the epidemiology of Bd is fundamentally different in Asia. It will be important to monitor the situation in Asia and distinguish between the two hypotheses. The former indicates that a Bd-caused amphibian epidemic may yet occur and the latter would mean that unique conditions of disease transmission may exist in Asia and warrant close study.

## Supporting Information

Table S1 **Infection status of** ***Batrachochytrium dendrobatidis*** **by locality.** Results of infection status for 298 localities shown with low infection and high infection positives grouped together here as “Bd Pos” and total number of sampled animals are provided. The 95% Bayesian credible intervals given in the last two columns.
(DOC)

Table S2 **Taxonomic groups sampled.** Families and genera of anura and caudata sampled in this study. The number of species in each genus is given as well as the sample size from each genus. The genera that tested positive for *Batrachochytrium dendrobatidis* in this study by qPCR or histology are indicated with an asterisk.
(DOCX)
