# Supplementary material for: Is Chytridiomycosis an Emerging Infectious Disease in Asia?
Source: PLoS One. 2011 Aug 16;6(8):e23179. doi: 10.1371/journal.pone.0023179 (PMC3156717; doi:10.1371/journal.pone.0023179)
Supplement: Table S1 — Infection status of Batrachochytrium dendrobatidis by locality. Results of infection status for 298 localities shown with low infection and high infection positives grouped together here as “Bd Pos” and total number of sampled animals are provided. The 95% Bayesian credible intervals given in the last two columns. (DOC) [file pone.0023179.s001.doc]

**Table S1 Infection status of *Batrachochytrium dendrobatidis* by locality**

| **Country** | **Province** | **Loc ID** | **Latitude (°N)** | **Longitude (°E)** | **Bd Pos** | **Total** | **95% CI lower** | **95% CI upper** |
| --- | --- | --- | --- | --- | --- | --- | --- | --- |
| Cambodia | Mondolkiri | 30 | 12.13336667 | 106.9037333 | 0 | 36 | 0.07 | 9.49 |
|  |  | 39 | 12.17905 | 106.8824667 | 0 | 2 | 0.84 | 70.76 |
|  |  | 53 | 12.2033 | 107.0190333 | 0 | 32 | 0.08 | 10.58 |
|  |  | 55 | 12.2096 | 107.0330167 | 0 | 36 | 0.07 | 9.49 |
|  | Pursat | 52 | 12.19646667 | 103.1191833 | 0 | 20 | 0.12 | 16.11 |
|  |  | 57 | 12.322 | 103.5138 | 0 | 30 | 0.08 | 11.22 |
|  |  | 58 | 12.42726667 | 103.8674833 | 0 | 4 | 0.51 | 52.18 |
|  | Ratanakiri | 65 | 14.18778333 | 106.9957333 | 0 | 44 | 0.06 | 7.87 |
|  |  | 66 | 14.19286667 | 106.9961167 | 0 | 94 | 0.03 | 3.81 |
|  |  | 67 |  | 106.9973 | 0 | 1 | 1.26 | 84.19 |
|  |  | 286 | 14.19693333 | 106.9973 | 0 | 1 | 1.26 | 84.19 |
|  |  | 68 | 14.19866667 | 106.9995 | 0 | 4 | 0.51 | 52.18 |
|  |  | 69 | 14.19895 | 106.9957333 | 0 | 1 | 1.26 | 84.19 |
|  |  | 70 |  | 106.99955 | 0 | 1 | 1.26 | 84.19 |
|  |  | 71 | 14.20795 | 107.00455 | 0 | 2 | 0.84 | 70.76 |
|  |  | 72 | 14.20905556 | 107.0015833 | 0 | 2 | 0.84 | 70.76 |
|  |  | 73 | 14.21732937 | 107.0145827 | 0 | 1 | 1.26 | 84.19 |
|  |  | 75 | 14.23593333 | 106.9822333 | 0 | 17 | 0.14 | 18.53 |
|  |  | 76 |  | 106.9973 | 0 | 1 | 1.26 | 84.19 |
|  |  | 77 | 14.23813333 | 106.9815833 | 0 | 8 | 0.28 | 33.63 |
|  | Stung Treng | 59 | 13.0748 | 105.80725 | 0 | 22 | 0.11 | 14.82 |
|  |  | 60 | 13.09123333 | 105.8142667 | 0 | 10 | 0.23 | 28.49 |
|  |  | 61 | 13.09158333 | 105.8128333 | 0 | 2 | 0.84 | 70.76 |
|  |  | 62 | 13.10716667 | 105.8137167 | 0 | 12 | 0.19 | 24.71 |
| China | Guizhou | 161 | 26.36601667 | 108.1989333 | 0 | 1 | 1.26 | 84.19 |
|  |  | 162 | 26.36666667 | 108.2066833 | 0 | 1 | 1.26 | 84.19 |
|  |  | 163 | 26.36685 | 108.20565 | 0 | 2 | 0.84 | 70.76 |
|  |  | 164 | 26.36766667 | 108.1552667 | 0 | 1 | 1.26 | 84.19 |
|  |  | 165 | 26.36813333 | 108.1745 | 0 | 1 | 1.26 | 84.19 |
|  |  | 166 | 26.36815 | 108.1870667 | 0 | 1 | 1.26 | 84.19 |
|  |  | 289 | 26.36816667 | 108.1870667 | 0 | 2 | 0.84 | 70.76 |
|  |  | 167 | 26.36893333 | 108.1783333 | 0 | 1 | 1.26 | 84.19 |
|  |  | 168 | 26.37035 | 108.19485 | 0 | 2 | 0.84 | 70.76 |
|  |  | 169 | 26.37035 | 108.1948833 | 0 | 1 | 1.26 | 84.19 |
|  |  | 170 | 26.3723 | 108.1941 | 0 | 1 | 1.26 | 84.19 |
|  |  | 171 | 26.3723 | 108.2441 | 0 | 1 | 1.26 | 84.19 |
|  |  | 172 | 26.37291667 | 108.2074833 | 0 | 1 | 1.26 | 84.19 |
|  |  | 173 | 26.37326667 | 108.20105 | 0 | 1 | 1.26 | 84.19 |
|  |  | 174 | 26.37338333 | 108.1916167 | 0 | 1 | 1.26 | 84.19 |
|  |  | 175 | 26.37366667 | 108.1989167 | 0 | 1 | 1.26 | 84.19 |
|  |  | 176 | 26.37373333 | 108.1953 | 0 | 1 | 1.26 | 84.19 |
|  |  | 177 | 26.374 | 108.1908 | 0 | 1 | 1.26 | 84.19 |
|  |  | 178 | 26.3740667 | 108.1993833 | 0 | 1 | 1.26 | 84.19 |
|  |  | 179 | 26.37425 | 108.1932 | 0 | 1 | 1.26 | 84.19 |
|  |  | 180 | 26.37441667 | 108.25395 | 0 | 1 | 1.26 | 84.19 |
|  |  | 181 | 26.37443333 | 108.2539667 | 0 | 1 | 1.26 | 84.19 |
|  |  | 182 | 26.37445 | 108.2539833 | 0 | 1 | 1.26 | 84.19 |
|  |  | 183 | 26.37446667 | 108.254 | 0 | 1 | 1.26 | 84.19 |
|  |  | 184 | 26.37448333 | 108.2540167 | 0 | 1 | 1.26 | 84.19 |
|  |  | 185 | 26.3745 | 108.2540333 | 0 | 1 | 1.26 | 84.19 |
|  |  | 186 | 26.37451667 | 108.25405 | 0 | 1 | 1.26 | 84.19 |
|  |  | 187 | 26.37453333 | 108.2540667 | 0 | 1 | 1.26 | 84.19 |
|  |  | 190 | 26.37456667 | 108.1932667 | 0 | 1 | 1.26 | 84.19 |
|  |  | 191 | 26.37475 | 108.20015 | 0 | 1 | 1.26 | 84.19 |
|  |  | 192 | 26.37493333 | 108.19515 | 0 | 1 | 1.26 | 84.19 |
|  |  | 193 | 26.37528333 | 108.2105333 | 0 | 1 | 1.26 | 84.19 |
|  |  | 194 | 26.37585 | 108.2006667 | 0 | 1 | 1.26 | 84.19 |
|  |  | 195 | 26.37605 | 108.19445 | 0 | 1 | 1.26 | 84.19 |
|  |  | 196 | 26.37705 | 108.2022333 | 0 | 1 | 1.26 | 84.19 |
|  |  | 188 | 26.37715 | 108.2022333 | 0 | 1 | 1.26 | 84.19 |
|  |  | 197 | 26.37951667 | 108.1916333 | 0 | 1 | 1.26 | 84.19 |
|  |  | 198 | 26.37961667 | 108.19175 | 0 | 1 | 1.26 | 84.19 |
|  |  | 199 | 26.38108333 | 108.1862833 | 0 | 1 | 1.26 | 84.19 |
|  |  | 200 | 26.3811 | 108.1862667 | 0 | 2 | 0.84 | 70.76 |
|  |  | 201 | 26.3826 | 108.1992 | 0 | 1 | 1.26 | 84.19 |
|  |  | 202 | 26.38398333 | 108.1985167 | 0 | 2 | 0.84 | 70.76 |
|  |  | 189 | 26.3841 | 108.1985167 | 0 | 1 | 1.26 | 84.19 |
|  |  | 203 | 26.3841 | 108.1986833 | 0 | 1 | 1.26 | 84.19 |
|  |  | 204 | 26.39668333 | 108.23755 | 0 | 1 | 1.26 | 84.19 |
|  |  | 235 | 27.82421667 | 108.7410667 | 0 | 3 | 0.63 | 60.24 |
|  |  | 241 | 27.85258333 | 108.75915 | 0 | 4 | 0.51 | 52.18 |
|  |  | 242 | 27.8581 | 108.7564167 | 0 | 2 | 0.84 | 70.76 |
|  |  | 294 | 27.85876667 | 108.7432667 | 0 | 2 | 0.84 | 70.76 |
|  |  | 243 | 27.86873333 | 108.74095 | 0 | 1 | 1.26 | 84.19 |
|  |  | 244 | 27.86905 | 108.7407 | 0 | 1 | 1.26 | 84.19 |
|  |  | 245 | 27.86988333 | 108.74005 | 0 | 1 | 1.26 | 84.19 |
|  |  | 246 | 27.87013333 | 108.7395333 | 0 | 2 | 0.84 | 70.76 |
|  |  | 247 | 27.87035 | 108.7397 | 0 | 1 | 1.26 | 84.19 |
|  |  | 248 | 27.87085 | 108.73935 | 0 | 1 | 1.26 | 84.19 |
|  |  | 251 | 27.88506667 | 108.7245333 | 0 | 1 | 1.26 | 84.19 |
|  |  | 252 | 27.88536667 | 108.7227167 | 0 | 1 | 1.26 | 84.19 |
|  |  | 254 | 27.88803333 | 108.7203 | 0 | 1 | 1.26 | 84.19 |
|  |  | 255 | 27.88845 | 108.7407333 | 0 | 6 | 0.36 | 40.96 |
|  |  | 256 | 27.88858333 | 108.71965 | 0 | 7 | 0.32 | 36.94 |
|  |  | 295 | 27.88901667 | 108.7709167 | 0 | 1 | 1.26 | 84.19 |
|  |  | 257 | 27.89075 | 108.7233667 | 0 | 7 | 0.32 | 36.94 |
|  |  | 258 | 27.8912 | 108.7757833 | 0 | 1 | 1.26 | 84.19 |
|  |  | 259 | 27.89266667 | 108.7240833 | 0 | 1 | 1.26 | 84.19 |
|  |  | 260 | 27.90233333 | 108.7182833 | 0 | 1 | 1.26 | 84.19 |
|  |  | 261 | 27.90283333 | 108.7553167 | 0 | 1 | 1.26 | 84.19 |
|  |  | 262 | 27.90351667 | 108.7206667 | 0 | 1 | 1.26 | 84.19 |
|  |  | 263 | 27.90371667 | 108.7220333 | 0 | 2 | 0.84 | 70.76 |
|  |  | 264 | 27.90398333 | 108.7211167 | 0 | 1 | 1.26 | 84.19 |
|  | Yunnan | 152 | 24.01755 | 99.68568333 | 0 | 5 | 0.42 | 45.93 |
|  |  | 153 | 24.01755 | 99.6857 | 0 | 1 | 1.26 | 84.19 |
|  |  | 154 | 24.01755 | 99.68571667 | 0 | 2 | 0.84 | 70.76 |
|  |  | 155 | 24.02593333 | 99.67338333 | 0 | 14 | 0.17 | 21.8 |
|  |  | 156 | 24.02665 | 99.67225 | 0 | 4 | 0.51 | 52.18 |
|  |  | 157 | 24.12876667 | 99.7268 | 0 | 10 | 0.23 | 28.49 |
|  |  | 158 | 24.1896 | 99.51623333 | 0 | 4 | 0.51 | 52.18 |
|  |  | 159 | 24.19323333 | 99.64476667 | 0 | 4 | 0.51 | 52.18 |
|  |  | 288 | 24.19333333 | 99.64381667 | 0 | 11 | 0.21 | 26.46 |
|  |  | 160 | 24.19333333 | 99.64383333 | 0 | 1 | 1.26 | 84.19 |
|  |  | 205 | 27.68833333 | 98.30411111 | 0 | 1 | 1.26 | 84.19 |
|  |  | 206 | 27.68855556 | 98.28661111 | 0 | 1 | 1.26 | 84.19 |
|  |  | 207 | 27.68880556 | 98.28416667 | 0 | 3 | 0.63 | 60.24 |
|  |  | 208 | 27.68963889 | 98.30488889 | 0 | 2 | 0.84 | 70.76 |
|  |  | 209 | 27.69016667 | 98.27911111 | 0 | 1 | 1.26 | 84.19 |
|  |  | 210 | 27.69016667 | 98.28355556 | 0 | 5 | 0.42 | 45.93 |
|  |  | 211 | 27.69119444 | 98.28244444 | 0 | 1 | 1.26 | 84.19 |
|  |  | 212 | 27.69491667 | 98.27836111 | 0 | 1 | 1.26 | 84.19 |
|  |  | 213 | 27.71444444 | 98.33813889 | 0 | 1 | 1.26 | 84.19 |
|  |  | 214 | 27.7365 | 98.35077778 | 0 | 2 | 0.84 | 70.76 |
|  |  | 215 | 27.73755556 | 98.35025 | 0 | 2 | 0.84 | 70.76 |
|  |  | 290 | 27.74081 | 98.35105 | 0 | 3 | 0.63 | 60.24 |
|  |  | 216 | 27.74530556 | 98.34952778 | 0 | 1 | 1.26 | 84.19 |
|  |  | 291 | 27.74725 | 98.34925 | 0 | 1 | 1.26 | 84.19 |
|  |  | 217 | 27.74873 | 98.34912 | 0 | 6 | 0.36 | 40.96 |
|  |  | 218 | 27.76213889 | 98.34566667 | 0 | 4 | 0.51 | 52.18 |
|  |  | 292 | 27.763 | 98.34266667 | 0 | 2 | 0.84 | 70.76 |
|  |  | 219 | 27.76461111 | 98.33980556 | 0 | 2 | 0.84 | 70.76 |
|  |  | 220 | 27.76525 | 98.33930556 | 0 | 2 | 0.84 | 70.76 |
|  |  | 221 | 27.76683333 | 98.33825 | 0 | 1 | 1.26 | 84.19 |
|  |  | 222 | 27.76886111 | 98.33808333 | 0 | 1 | 1.26 | 84.19 |
|  |  | 223 | 27.77091667 | 98.33680556 | 0 | 10 | 0.23 | 28.49 |
|  |  | 224 | 27.77111111 | 98.33694444 | 0 | 1 | 1.26 | 84.19 |
|  |  | 225 | 27.77141667 | 98.33669444 | 0 | 1 | 1.26 | 84.19 |
|  |  | 226 | 27.77469444 | 98.33544444 | 0 | 2 | 0.84 | 70.76 |
|  |  | 227 | 27.77711111 | 98.33297222 | 0 | 6 | 0.36 | 40.96 |
|  |  | 228 | 27.78255556 | 98.33327778 | 0 | 1 | 1.26 | 84.19 |
|  |  | 229 | 27.78455556 | 98.33294444 | 0 | 2 | 0.84 | 70.76 |
|  |  | 230 | 27.78586111 | 98.33291667 | 0 | 8 | 0.28 | 33.63 |
|  |  | 293 | 27.78602778 | 98.33291667 | 0 | 1 | 1.26 | 84.19 |
|  |  | 231 | 27.78711111 | 98.33255556 | 0 | 1 | 1.26 | 84.19 |
|  |  | 232 | 27.78858333 | 98.33194444 | 0 | 1 | 1.26 | 84.19 |
|  |  | 233 | 27.78863889 | 98.33183333 | 0 | 1 | 1.26 | 84.19 |
|  |  | 234 | 27.78958333 | 98.33072222 | 0 | 2 | 0.84 | 70.76 |
|  |  | 236 | 27.82766667 | 98.32725 | 0 | 1 | 1.26 | 84.19 |
|  |  | 237 | 27.83269444 | 98.33286111 | 0 | 4 | 0.51 | 52.18 |
|  |  | 238 | 27.83569444 | 98.333 | 0 | 1 | 1.26 | 84.19 |
|  |  | 239 | 27.83638889 | 98.33241667 | 0 | 2 | 0.84 | 70.76 |
|  |  | 240 | 27.84122222 | 98.32883333 | 0 | 6 | 0.36 | 40.96 |
|  |  | 249 | 27.87402778 | 98.33638889 | 0 | 1 | 1.26 | 84.19 |
|  |  | 250 | 27.88411111 | 98.34336111 | 0 | 1 | 1.26 | 84.19 |
|  |  | 253 | 27.88558333 | 98.3435 | 0 | 1 | 1.26 | 84.19 |
| Indonesia | Sulawesi | 15 | 1.66352 | 121.88001 | 0 | 27 | 0.09 | 12.34 |
|  | Sulawesi | 14 | 1.42101 | 121.13778 | 0 | 34 | 0.07 | 10 |
|  | West Java | 2 | -6.906944444 | 106.82625 | 0 | 28 | 0.09 | 11.94 |
|  |  | 3 | -6.87275 | 106.5187222 | 0 | 78 | 0.03 | 4.56 |
|  |  | 4 | -6.853361111 | 106.5010611 | 0 | 54 | 0.05 | 6.49 |
|  |  | 5 | -6.850969444 | 106.9168444 | 0 | 46 | 0.05 | 7.55 |
|  |  | 6 | -6.741261111 | 106.6496278 | 6 | 60 | 4.74 | 20.19 |
|  |  | 7 | -6.717702778 | 106.8272222 | 0 | 48 | 0.05 | 7.25 |
|  |  | 8 | -6.659916667 | 106.9480139 | 0 | 160 | 0.02 | 2.27 |
|  |  | 9 | -6.655030556 | 106.7502694 | 0 | 66 | 0.04 | 5.36 |
|  |  | 10 | -6.564111111 | 106.7356111 | 0 | 80 | 0.03 | 4.45 |
| Kazakhstan | Almaty | 280 | 43.2612 | 76.9654 | 0 | 4 | 0.51 | 52.18 |
| Kyrgyzstan | Bishkek | 277 | 42.681 | 74.657 | 6 | 6 | 59.04 | 99.64 |
|  |  | 278 | 42.728 | 74.648 | 2 | 2 | 29.24 | 99.16 |
|  |  | 279 | 42.794 | 74.76 | 1 | 1 | 15.81 | 98.74 |
| Laos | Vientiane Prefecture | 128 | 18.040833 | 102.6372 | 0 | 23 | 0.11 | 14.25 |
|  | Huaphahn | 140 | 20.23253 | 103.21084 | 0 | 66 | 0.04 | 5.36 |
|  |  | 141 | 20.25995 | 103.19661 | 0 | 13 | 0.18 | 23.16 |
|  | Khammouan | 125 | 17.62527778 | 105.7173056 | 0 | 15 | 0.16 | 20.59 |
|  |  | 126 | 17.62536111 | 105.7186944 | 0 | 11 | 0.21 | 26.46 |
|  |  | 127 | 17.64433333 | 105.73675 | 0 | 101 | 0.02 | 3.55 |
|  | Luang Namtha | 143 | 20.86888889 | 101.0552778 | 4 | 111 | 1.47 | 8.89 |
|  | Savannakhet | 87 | 16.94211111 | 106.0585 | 0 | 7 | 0.32 | 36.94 |
|  |  | 88 | 16.94347222 | 106.0574167 | 0 | 2 | 0.84 | 70.76 |
|  |  | 89 | 16.95041667 | 105.8967778 | 0 | 15 | 0.16 | 20.59 |
|  |  | 90 | 16.95652778 | 106.0676667 | 0 | 27 | 0.09 | 12.34 |
|  |  | 91 | 16.95863889 | 106.0499722 | 0 | 2 | 0.84 | 70.76 |
|  |  | 92 | 16.95872222 | 106.0682778 | 0 | 1 | 1.26 | 84.19 |
|  |  | 93 | 16.95944444 | 106.0494722 | 0 | 3 | 0.63 | 60.24 |
|  |  | 94 | 16.96 | 106.0678611 | 0 | 2 | 0.84 | 70.76 |
|  |  | 95 | 16.96002778 | 106.0471111 | 0 | 5 | 0.42 | 45.93 |
|  |  | 96 | 16.96102778 | 106.0473611 | 0 | 3 | 0.63 | 60.24 |
|  |  | 97 | 16.96238889 | 106.0485278 | 0 | 2 | 0.84 | 70.76 |
|  |  | 98 | 16.96316667 | 106.0466111 | 0 | 25 | 0.1 | 13.23 |
|  |  | 99 | 16.965 | 105.8118056 | 0 | 2 | 0.84 | 70.76 |
|  |  | 100 | 16.96505556 | 105.8122222 | 0 | 2 | 0.84 | 70.76 |
|  |  | 101 | 16.96705556 | 105.8136944 | 0 | 7 | 0.32 | 36.94 |
|  |  | 102 | 16.96791667 | 105.8167778 | 0 | 1 | 1.26 | 84.19 |
|  |  | 103 | 16.96916667 | 105.8117222 | 0 | 7 | 0.32 | 36.94 |
|  |  | 104 | 16.97183333 | 106.1665833 | 0 | 11 | 0.21 | 26.46 |
|  |  | 105 | 16.97647222 | 106.1721111 | 0 | 1 | 1.26 | 84.19 |
|  |  | 106 | 16.98022222 | 105.8065556 | 0 | 8 | 0.28 | 33.63 |
|  |  | 107 | 16.98838889 | 105.8098611 | 0 | 4 | 0.51 | 52.18 |
|  |  | 108 | 17.01180556 | 106.2201389 | 0 | 6 | 0.36 | 40.96 |
|  |  | 109 | 17.02261111 | 106.1676667 | 0 | 2 | 0.84 | 70.76 |
|  |  | 110 | 17.03013889 | 106.0178056 | 0 | 1 | 1.26 | 84.19 |
|  |  | 116 | 17.04297222 | 106.1250278 | 0 | 18 | 0.13 | 17.65 |
|  |  | 118 | 17.04430556 | 106.1220278 | 0 | 2 | 0.84 | 70.76 |
|  |  | 120 | 17.04444444 | 106.1262222 | 0 | 12 | 0.19 | 24.71 |
|  |  | 122 | 17.04786111 | 106.1278889 | 0 | 10 | 0.23 | 28.49 |
|  |  | 124 | 17.05272222 | 106.13875 | 0 | 3 | 0.63 | 60.24 |
| Malaysia | Sabah | 18 | 6.045991667 | 116.5973861 | 0 | 3 | 0.63 | 60.24 |
|  |  | 17 | 6.008611111 | 116.5427694 | 2 | 51 | 1.21 | 13.21 |
|  |  | 16 | 5.956191667 | 116.6665639 | 0 | 22 | 0.11 | 14.82 |
| Mongolia | Arkhangay | 284 | 47.81325 | 101.547083 | 0 | 4 | 0.51 | 52.18 |
|  | Bayanhongor | 281 | 45.623661 | 99.261303 | 0 | 1 | 1.26 | 84.19 |
|  | Govi Altay | 282 | 45.707 | 97.221806 | 0 | 12 | 0.19 | 24.71 |
|  |  | 283 | 45.7139 | 97.197231 | 0 | 6 | 0.36 | 40.96 |
| Myanmar | Yangon | 111 | 17.04019444 | 96.09738889 | 0 | 10 | 0.23 | 28.49 |
|  |  | 112 | 17.041 | 96.09830556 | 0 | 4 | 0.51 | 52.18 |
|  |  | 113 | 17.04102778 | 96.09738889 | 0 | 3 | 0.63 | 60.24 |
|  |  | 114 | 17.04141667 | 96.09644444 | 0 | 1 | 1.26 | 84.19 |
|  |  | 115 | 17.04286111 | 96.10397222 | 0 | 3 | 0.63 | 60.24 |
|  |  | 117 | 17.04430556 | 96.10494444 | 0 | 11 | 0.21 | 26.46 |
|  |  | 119 | 17.04438889 | 96.10655556 | 0 | 13 | 0.18 | 23.16 |
|  |  | 121 | 17.04619444 | 96.09336111 | 0 | 5 | 0.42 | 45.93 |
|  |  | 123 | 17.04813889 | 96.09483333 | 0 | 12 | 0.19 | 24.71 |
| Pakistan | Pishin | 265 | 30.61333333 | 67.02833333 | 0 | 5 | 0.42 | 45.93 |
| Papua New Guinea | Port Moresby | 1 | -9.446666667 | 147.185 | 0 | 1 | 1.26 | 84.19 |
|  | West New Britain | 287 | -5.455 | 150.1083333 | 0 | 4 | 0.51 | 52.18 |
|  |  | 11 | -5.40833333 | 151.1383333 | 0 | 65 | 0.04 | 5.44 |
|  |  | 12 | -5.3083333 | 151.075 | 0 | 1 | 1.26 | 84.19 |
|  |  | 13 | -5.27 | 151.4466666 | 0 | 2 | 0.84 | 70.76 |
| Philippines | Barangay | 79 | 14.32733333 | 120.9573333 | 0 | 22 | 0.11 | 14.82 |
|  | Batanes | 142 | 20.47 | 121.991 | 0 | 11 | 0.21 | 26.46 |
|  | Leyte | 25 | 10.67789167 | 124.6425 | 0 | 16 | 0.15 | 19.51 |
|  |  | 26 | 10.74388 | 124.8 | 0 | 4 | 0.51 | 52.18 |
|  | Camarines Norte | 63 | 14.03936667 | 122.78655 | 1 | 79 | 0.3 | 6.77 |
|  |  | 64 | 14.158325 | 121.2227972 | 0 | 7 | 0.32 | 36.94 |
|  | Cavite | 78 | 14.32496944 | 120.9597361 | 0 | 123 | 0.02 | 2.93 |
|  | Zamboanga del Sur | 24 | 8.222388889 | 125.932444 | 0 | 15 | 0.16 | 20.59 |
|  |  | 21 | 6.977067 | 122.067383 | 0 | 49 | 0.05 | 7.11 |
|  |  | 22 | 6.99223333 | 122.0604333 | 0 | 3 | 0.63 | 60.24 |
|  | Batangas | 74 | 14.23233333 | 120.6575 | 33 | 42 | 63.96 | 88.24 |
|  | Aurora | 86 | 15.742333 | 121.5925 | 0 | 23 | 0.11 | 14.25 |
|  | Quezon | 80 | 14.8671 | 121.9437833 | 0 | 10 | 0.23 | 28.49 |
| South Korea | Chungcheongbuk | 276 | 36.53896667 | 127.8380333 | 0 | 1 | 1.26 | 84.19 |
|  |  | 275 | 36.52723333 | 127.8576 | 0 | 2 | 0.84 | 70.76 |
|  | Busan | 296 | 35.3307 | 129.2890333 | 0 | 1 | 1.26 | 84.19 |
|  |  | 267 | 35.33 | 129.2883333 | 0 | 3 | 0.63 | 60.24 |
|  | Daejeon | 272 | 36.22506667 | 127.34315 | 0 | 6 | 0.36 | 40.96 |
|  | Jeollabuk | 270 | 35.76618333 | 127.4106167 | 0 | 1 | 1.26 | 84.19 |
|  | Jeolanam | 268 | 35.40473333 | 126.8997 | 0 | 1 | 1.26 | 84.19 |
|  |  | 269 | 35.40473333 | 127.8997 | 0 | 1 | 1.26 | 84.19 |
|  | Gyeongsangnam | 266 | 35.23061667 | 127.6460167 | 0 | 1 | 1.26 | 84.19 |
|  | Jeolanam | 271 | 36.18038333 | 127.5717 | 0 | 2 | 0.84 | 70.76 |
|  | Chungcheongnam | 273 | 36.22668333 | 127.50535 | 1 | 1 | 15.81 | 98.74 |
|  |  | 274 | 36.30053333 | 127.2029 | 0 | 2 | 0.84 | 70.76 |
|  |  | 285 | 36.06333333 | 127.3951667 | 0 | 1 | 1.26 | 84.19 |
|  |  | 297 | 36.05151667 | 127.4771167 | 1 | 4 | 5.27 | 71.64 |
| Sri Lanka | Central | 19 | 6.843333333 | 80.67777778 | 9 | 86 | 5.65 | 18.73 |
|  |  | 23 | 7.253644444 | 80.60173889 | 0 | 11 | 0.21 | 26.46 |
|  | Uva | 20 | 6.884558333 | 80.80136111 | 1 | 20 | 1.17 | 23.82 |
| Vietnam | Dong Nai | 27 | 11.08286111 | 107.3970278 | 0 | 13 | 0.18 | 23.16 |
|  |  | 28 | 11.10361111 | 107.4085556 | 0 | 14 | 0.17 | 21.8 |
|  | Ha Tinh | 129 | 18.54177778 | 105.7107222 | 0 | 16 | 0.15 | 19.51 |
|  | Khanh Hoa | 44 | 12.19105556 | 108.7170278 | 0 | 14 | 0.17 | 21.8 |
|  |  | 45 | 12.19180556 | 108.7176389 | 0 | 19 | 0.13 | 16.84 |
|  |  | 47 | 12.19225 | 108.7896667 | 0 | 1 | 1.26 | 84.19 |
|  | Lam Dong | 29 | 12.12847222 | 108.68475 | 0 | 1 | 1.26 | 84.19 |
|  |  | 31 | 12.16225 | 108.6650833 | 0 | 9 | 0.25 | 30.85 |
|  |  | 32 | 12.16344444 | 108.6629167 | 0 | 21 | 0.12 | 15.44 |
|  |  | 33 | 12.17352778 | 108.6705 | 0 | 13 | 0.18 | 23.16 |
|  |  | 34 | 12.17536111 | 108.7004167 | 0 | 31 | 0.08 | 10.89 |
|  |  | 35 | 12.17558333 | 108.7005556 | 0 | 1 | 1.26 | 84.19 |
|  |  | 36 | 12.17558333 | 108.70695 | 0 | 1 | 1.26 | 84.19 |
|  |  | 37 | 12.17558333 | 108.7115833 | 0 | 18 | 0.13 | 17.65 |
|  |  | 38 | 12.17638889 | 108.69825 | 0 | 18 | 0.13 | 17.65 |
|  |  | 40 | 12.18252778 | 108.6790833 | 0 | 3 | 0.63 | 60.24 |
|  |  | 42 | 12.18644444 | 108.714861 | 0 | 23 | 0.11 | 14.25 |
|  |  | 41 | 12.18644444 | 108.7148611 | 3 | 63 | 1.73 | 13.09 |
|  |  | 43 | 12.19008333 | 108.7816667 | 0 | 1 | 1.26 | 84.19 |
|  |  | 46 | 12.19225 | 108.7149444 | 0 | 4 | 0.51 | 52.18 |
|  |  | 48 | 12.19258333 | 108.7115556 | 0 | 3 | 0.63 | 60.24 |
|  |  | 49 | 12.19261111 | 108.7115833 | 3 | 14 | 7.79 | 48.09 |
|  |  | 50 | 12.19313889 | 108.7126944 | 1 | 1 | 15.81 | 98.74 |
|  |  | 51 | 12.19333333 | 108.712 | 0 | 3 | 0.63 | 60.24 |
|  |  | 54 | 12.20791667 | 108.69325 | 0 | 2 | 0.84 | 70.76 |
|  |  | 56 | 12.21602778 | 108.7133056 | 0 | 1 | 1.26 | 84.19 |
|  | Lao Cai | 144 | 21.9722 | 104.26175 | 0 | 10 | 0.23 | 28.49 |
|  |  | 145 | 21.97455 | 104.2648667 | 0 | 18 | 0.13 | 17.65 |
|  |  | 146 | 21.98741667 | 104.26575 | 0 | 15 | 0.16 | 20.59 |
|  |  | 147 | 21.98741667 | 104.2657667 | 0 | 1 | 1.26 | 84.19 |
|  |  | 148 | 21.98943333 | 104.2629 | 0 | 10 | 0.23 | 28.49 |
|  |  | 149 | 21.9915 | 104.2703167 | 0 | 26 | 0.09 | 12.77 |
|  |  | 150 | 21.99153333 | 104.27025 | 0 | 1 | 1.26 | 84.19 |
|  |  | 151 | 21.99153333 | 104.2703167 | 0 | 1 | 1.26 | 84.19 |
|  | Nghe An | 130 | 18.80130556 | 104.8687778 | 0 | 4 | 0.51 | 52.18 |
|  |  | 131 | 18.80175 | 104.8731944 | 0 | 5 | 0.42 | 45.93 |
|  |  | 132 | 18.80211111 | 104.8691389 | 0 | 18 | 0.13 | 17.65 |
|  |  | 133 | 18.80211111 | 104.8704722 | 0 | 4 | 0.51 | 52.18 |
|  |  | 134 | 18.80266667 | 104.8691389 | 0 | 6 | 0.36 | 40.96 |
|  |  | 135 | 18.80366667 | 104.8689444 | 0 | 7 | 0.32 | 36.94 |
|  |  | 136 | 18.82472222 | 104.8568889 | 0 | 15 | 0.16 | 20.59 |
|  |  | 137 | 18.82488889 | 104.857 | 0 | 3 | 0.63 | 60.24 |
|  |  | 298 | 18.82869444 | 104.8468889 | 0 | 2 | 0.84 | 70.76 |
|  |  | 138 | 18.83033333 | 104.8468889 | 0 | 13 | 0.18 | 23.16 |
|  |  | 139 | 18.83141667 | 104.8675278 | 0 | 1 | 1.26 | 84.19 |
|  | Quang Nam | 81 | 15.26805 | 107.7545667 | 0 | 18 | 0.13 | 17.65 |
|  |  | 82 | 15.27243333 | 107.759 | 0 | 6 | 0.36 | 40.96 |
|  |  | 83 | 15.27393333 | 107.76015 | 0 | 6 | 0.36 | 40.96 |
|  |  | 84 | 15.27843333 | 107.7362 | 0 | 22 | 0.11 | 14.82 |
|  |  | 85 | 15.3219 | 107.7984667 | 0 | 10 | 0.23 | 28.49 |
