# Supplementary material for: Is Chytridiomycosis an Emerging Infectious Disease in Asia?
Source: PLoS One. 2011 Aug 16;6(8):e23179. doi: 10.1371/journal.pone.0023179 (PMC3156717; doi:10.1371/journal.pone.0023179)
Supplement: Table S2 — Taxonomic groups sampled. Families and genera of anura and caudata sampled in this study. The number of species in each genus is given as well as the sample size from each genus. The genera that tested positive for Batrachochytrium dendrobatidis in this study by qPCR or histology are indicated with an asterisk. (DOCX) [file pone.0023179.s002.docx]

**Table S2** **Taxonomic groups sampled**

| **Order** | **Family** | **Genus** | **Species (No.)** | **Samples (No.)** |
| --- | --- | --- | --- | --- |
| Anura | Bombinidae | *Bombina* | 1 | 8 |
|  | Bufonidae | *Ansonia* | 5 | 12 |
|  |  | *Bufo* | 4 | 11 |
|  |  | *Duttaphrynus* | 1 | 33 |
|  |  | *Ingerophrynus* | 4 | 147 |
|  |  | *Oreophryne* | 1 | 1 |
|  |  | *Pelophryne* | 1 | 2 |
|  |  | *Phrynoidis* | 1 | 28 |
|  |  | *Pseudepidalea** | 4 | 41 |
|  |  | unidentified | 1 | 1 |
|  | Ceratobatrachidae | *Platymantis* | 12 | 75 |
|  | Dicroglossidae | *Fejervarya* | 4 | 391 |
|  |  | *Hoplobatrachus* | 1 | 59 |
|  |  | *Limnonectes** | 15 | 438 |
|  |  | *Nanorana* | 3 | 61 |
|  |  | *Occidozyga** | 4 | 78 |
|  |  | *Paa* | 4 | 43 |
|  | Hylidae | *Hyla** | 1 | 4 |
|  |  | *Litoria* | 1 | 6 |
|  | Megophryidae | *Brachytarsophrys* | 1 | 15 |
|  |  | *Leptobrachella* | 1 | 1 |
|  |  | *Leptobrachium* | 10 | 144 |
|  |  | *Leptolalax** | 8 | 79 |
|  |  | *Megophrys* | 5 | 15 |
|  |  | *Ophryophryne** | 6 | 97 |
|  |  | *Oreolalax* | 1 | 12 |
|  |  | *Xenophrys* | 4 | 114 |
|  | Microhylidae | *Kalophrynus* | 2 | 11 |
|  |  | *Kaloula* | 6 | 10 |
|  |  | *Metaphrynella* | 1 | 1 |
|  |  | *Microhyla* | 12 | 71 |
|  |  | *Micryletta* | 1 | 5 |
|  | Nyctibatrachidae | *Lankanectes* | 1 | 7 |
|  | Ranidae | *Amolops* | 7 | 108 |
|  |  | *Huia* | 1 | 58 |
|  |  | *Hylarana** | 20 | 377 |
|  |  | *Lithobates* | 1 | 10 |
|  |  | *Meristogenys* | 2 | 16 |
|  |  | *Odorrana* | 14 | 210 |
|  |  | *Pelophylax* | 1 | 10 |
|  |  | *Rana* | 6 | 45 |
|  |  | *Staurois* | 2 | 10 |
|  |  | unidentified | 2 | 4 |
|  | Rhacophoridae | *Chirixalus* | 1 | 2 |
|  |  | *Feihyla* | 1 | 8 |
|  |  | *Kurixalus* | 3 | 28 |
|  |  | *Nyctixalus* | 1 | 2 |
|  |  | *Philautus** | 7 | 89 |
|  |  | *Pseudophilautus* | 3 | 64 |
|  |  | *Polypedates* | 3 | 110 |
|  |  | *Rhacophorus* | 17 | 132 |
|  |  | *Theloderma* | 2 | 6 |
|  | Unidentified | unidentified |  | 5 |
| Caudata | Hynobiidae | *Hynobius* | 3 | 6 |
|  |  | *Onychodactylus* | 1 | 2 |
|  | Plethodontidae | *Karsenia* | 1 | 3 |
|  | Salamandridae | *Pachytriton* | 1 | 4 |
|  |  | *Paramesotriton* | 3 | 31 |
|  |  | *Tylototriton* | 1 | 2 |
| Total |  |  | 242 | 3363 |
